# The presentation of neuroendocrine self‐peptides in the thymus: an essential event for individual life and vertebrate survival

**DOI:** 10.1111/nyas.14089

**Published:** 2019-04-22

**Authors:** Vincent Geenen, Charlotte Trussart, Hélène Michaux, Aymen Halouani, Hela Jaïdane, Caroline Collée, Chantal Renard, Marc Daukandt, Philippe Ledent, Henri Martens

**Affiliations:** ^1^ GIGA Institute University of Liège Liège‐Sart Tilman Belgium; ^2^ Faculty of Sciences and Faculty of Pharmacy University of Tunis El Manar Monastir Tunisia; ^3^ X‐Press Biologics Industrial Park of Milmort Liège Belgium

**Keywords:** self‐peptide, thymus, self‐tolerance, autoimmunity, reverse, tolerogenic, self‐vaccine

## Abstract

Confirming Burnet's early hypothesis, elimination of self‐reactive T cells in the thymus was demonstrated in the late 1980s, and an important question immediately arose about the nature of the self‐peptides expressed in the thymus. Many genes encoding neuroendocrine‐related and tissue‐restricted antigens (TRAs) are transcribed in thymic epithelial cells (TECs). They are then processed for presentation by proteins of the major histocompatibility complex (MHC) expressed by TECs and thymic dendritic cells. MHC presentation of self‐peptides in the thymus programs self‐tolerance by two complementary mechanisms: (1) negative selection of self‐reactive “forbidden” T cell clones starting already in fetal life, and (2) generation of self‐specific thymic regulatory T lymphocytes (tT_reg_ cells), mainly after birth. Many studies, including the discovery of the transcription factors autoimmune regulator (AIRE) and fasciculation and elongation protein zeta family zinc finger (FEZF2), have shown that a defect in thymus central self‐tolerance is the earliest event promoting autoimmunity. AIRE and FEZF2 control the level of transcription of many neuroendocrine self‐peptides and TRAs in the thymic epithelium. Furthermore, *AIRE* and *FEZF2* mutations are associated with the development of autoimmunity in peripheral organs. The discovery of the intrathymic presentation of self‐peptides has revolutionized our knowledge of immunology and is opening novel avenues for prevention/treatment of autoimmunity.

## Graphical abstract

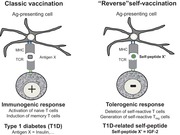

## Summary of thymus history

The word “thymus” first appeared in the manuscripts of Claudius Galen (129−around 216 A.D.) and was described as an excrescence having some morphological analogy with the leaf of the plant *Thymus cunila* (savory or “sarriette” in French). Galen thought that the thymus had no other function than providing protection between the sternum and superior vena cava. Galen also observed that the size of the thymus was larger in young animals and decreased with aging.^1^, ^2^

Over many centuries, the anatomy of the thymus was the essential focus of the studies on this organ until the Scottish embryologist John Beard (1858−1924), a pioneer of today's theory of cancer stem cells, wrote that he considered the thymus to be the source of some white blood cells in the body.^3^, ^4^

In 1890, the German anatomist Wilhelm Waldeyer (1836−1921) noticed that in elderly people islets of normal thymic tissue could be observed even after adipose involution of this organ. Jan‐August Hammar (Sweden, 1841−1946) also showed that, although thymic development is maximal at puberty, normal thymic tissue persists until advanced age. He observed that animal castration before puberty prevents thymic involution with age and that thymic hypoplasia is associated with pregnancy, undernourishment, and some infectious diseases. He showed that, conversely, thymic hyperplasia is associated with autoimmune Graves’ thyroid disease, Addison's adrenal insufficiency, myasthenia, and acromegaly.^5^ The thymus was then seen just as another glandular component of the endocrine system, and this assumption was reinforced by Hans Selye's observation of a severe thymic atrophy in stressful conditions activating the hypothalamic−pituitary−adrenal axis.^6^

The first immunological function of the thymus was discovered when French‐born Australian immunologist Jacques F.A.P. Miller showed that thymectomy performed in mice immediately after birth rendered them highly susceptible to infections and provoked their premature death. He also observed a marked lymphopenia in blood, spleen, and lymph nodes of these mice. These animals were also unable to reject a foreign skin graft, an essential hallmark of the immune response at that time. Miller concluded that the thymus was the organ responsible for the development of immunocompetent cells that constitute a specific cell population, thymus‐dependent (T) lymphocytes.^7^, ^8^ Despite the rightness of Miller's experiments and conclusions, the British immunologist Sir Peter Medawar (the 1960 Nobel Prize), regarded as the father of transplantation, still wrote in 1963: “We shall come to regard the presence of lymphocytes in the thymus as an evolutionary accident of no very great significance.”^9^

In the perspective of hematopoietic growth factors identified at that time, Miller advanced the hypothesis of one or several soluble thymic factors that would be responsible for driving T cell differentiation.^10^ Innumerable studies worldwide failed to demonstrate the existence of such thymus‐specific growth factor(s)/hormone(s) and it was never possible to apply the endocrine model to the communication between thymic epithelial cells (TECs) and thymocytes (T cells). The demonstration of a crucial role of the thymus during fetal life, as well as the absence of any pathogenicity resulting from thymectomy a few days after birth, reinforced the idea that the thymus was, if not a vestigial organ, at least an organ that quickly becomes useless, this being verified in human clinics: the DiGeorge congenital syndrome, which is the most common form of genetic microdeletion, associates, together with other defects, with the absence or hypoplasia of the thymus and severe immunodeficiency. On the other hand, children who have been thymectomized during surgical correction of a congenital cardiac defect do not suffer from any patent immune deficiency further in life.

## Immunological self‐tolerance

Since the foundation of immunology at the end of the 19th century, prediction of autotoxicity/autoimmunity has been intimately associated with the discovery of antitoxins/antibodies. As soon as in 1900, Paul Ehrlich (1854−1915, the 1908 Nobel Prize) proposed the aphorism “horror autotoxicus” to claim the impossibility that one organism could be attacked under normal conditions by its own cells in charge for its defenses. Ehrlich thought then that either structures or mechanisms should exist to avoid autotoxicity, and this should be of the highest importance for individual health and species survival.^11^ In the continuation of his revolutionary theory of clonal selection, the Australian virologist and immunologist Sir Frank Macfarlane Burnet (1899−1985), who shared the 1960 Nobel Prize with Medawar, introduced the term *tolerance* to characterize one of the cardinal properties of the adaptive immune system with diversity and memory. In marked contrast with Medawar's statement, during a conference at the University of London in 1962, Burnet prophesized: “If, as I think, the thymus is the site where occurs proliferation of lymphocytes in clones with precise immunological functions, we have also to consider another function: elimination or inhibition of clones with reactivity to self.”

Afterward, the molecular mechanisms responsible for the stochastic recombination of gene segments encoding the variable domains of the immunoglobulin B cell receptor for the antigen (BCR)^12^ and T cell receptor (TCR) for the antigen were elucidated.^13^, ^14^, ^15^ The fantastic lottery behind the generation of diversity in the adaptive immune system may produce more than 10^30^ cumulated TCR and BCR combinations, the majority of which are able to recognize self‐antigens. In normal conditions, however, the adaptive immune system does not aggress self and, for a long time, immunology was defined as the science of self−nonself discrimination. Burnet was actually speaking about immunology as the science of self and nonself recognition. Indeed, without “training,” lymphocytes are not able to discriminate between self‐ and nonself‐peptides. In 1987 and 1988, the research groups of Douarin,^16^ Kappler and Marrack,^17^ MacDonald,^18^ and von Boehmer^19^ demonstrated the validity of the theory of thymic clonal negative selection proposed by Burnet many years before. These independent studies clearly evidenced that education to self and establishment of T cell self‐tolerance is imperative even *before* the acquisition of T cell immunocompetence. Therefore, the thymus is not only responsible for the generation of diversity of the TCR repertoire, but is also primarily a cemetery for early T cells expressing a TCR specific for self‐antigens that are presented to differentiating T cells by proteins of the major histocompatibility complex (MHC) expressed by TECs and thymic dendritic cells (DCs).

In 1972, Richard Gershon (1932−1983) from Yale identified immunosuppressive cells regulating competent lymphocytes.^20^ After his premature death, his studies were pursued by Shimon Sakaguchi who, in a series of experiments, identified a novel key role for thymus‐derived T cells in the regulation of autoimmune responses.^21^, ^22^, ^23^ Indeed, mainly after birth, the thymus is the source of a new population of tT_reg_ cells that are able to inhibit in the peripheral self‐reactive T cells having escaped negative selection in the thymus. The Foxp3 transcription factor, through its expression in T_reg_ cells, is essential for the prevention of autoimmunity as shown in the human immune dysregulation, polyendocrinopathy, enteropathy, X‐linked (IPEX) syndrome, as well as in scurfy and *Foxp3^−/−^* mice.^24^, ^25^, ^26^ The generation of tT_reg_ cells also depends on the presentation of self‐antigens by thymic MHC proteins. How the same mechanism of MHC‐mediated self‐peptide presentation promotes CD4^+^ fates that are so distinct during thymic T cell differentiation (negative selection of self‐reactive T cells and generation of self‐specific tT_reg_ cells) is still a matter of active exploration.^27^

## A creative metaphor leading to a novel paradigm: from neuropeptides to neuroendocrine “self‐peptides”

Following these seminal studies about the powerful tolerogenic mechanisms occurring inside the thymus, the real nature of self‐peptides that are presented by thymic MHC proteins was then scrutinized. Previously, it was largely assumed that proteins circulating in the blood were captured somewhat “passively” in the thymus (by DCs and macrophages, mainly) and then presented to differentiating T lymphocytes during their transitory residence in this organ. In 1978, immunoreactive neurotensin (NT) and somatostatin were identified in the chicken thymus epithelium,^28^ but these observations were not followed further. In 1986, synthesis of biologically active and immunoreactive oxytocin (OT), in equimolar content with neurophysin, its binding protein derived from the same precursor, was discovered in human thymus extracts.^29^ In the thymus microenvironment, OT is synthesized by TECs and not by thymocytes.^30^ Following the metaphor of the “nursing” of newborns through OT galactagogue action, thymic “nurse” cells (TNCs) were shown to be a cortical TEC population synthesizing OT.^31^ Intrathymic of the oxytocin gene *(OXT)* transcription coincides with its expression in the hypothalamus.^32^ Furthermore, functional neurohypophysial receptors (OTR and V1b) are expressed by distinct thymic T cell subsets. After binding to these receptors, OT—much more than vasopressin (VP)—promotes phosphorylation of T cell tyrosine kinases closely implicated in focal adhesion, and this event could be implicated in the formation of immunological synapses between TECs and thymocytes.^33^ Neuropeptide Y (NPY), neurokinin A (NKA),^34^ and insulin‐like growth factor‐2 (IGF‐2)^35^, ^36^ were also found to be synthesized in the thymic epithelium. The repertoire of neuroendocrine‐related precursors is organized in such an economical way that one member per family is predominantly expressed in TECs: OT for the neurohypophysial family, NT for neuromedins, NKA for tachykinins, and IGF‐2 for the insulin family.^37^, ^38^ TECs were proposed to exhibit a more “promiscuous” promoter use than other peripheral tissues.^39^

Most importantly, in the thymus, the processing of OT precursor does not lead to classic neurosecretion as established a long time ago for the hypothalamo−neurohypophysial axis. Thymic OT is not located in classic secretory granules, but is diffuse within TEC/TNC cytosol, in large clear vacuoles, and around perimembranous space.^40^ Actually, OT precursor is processed for the presentation of OT peptide by thymic MHC class I (MHC‐I) molecules, and the neurophysin domain of OT precursor is associated in this presentation through an MHC‐I−neurophysin 55 kD hybrid molecule.^41^ The biochemical mechanism underlying formation of this hybrid protein remains to be specified but, nevertheless, these data suggest an analogy of neurophysin function between, on the one hand, the transport of the neurohormone OT along hypothalamo−neurohypophysial neurons to nerve endings in posterior pituitary and, on the other hand, the intrathymic presentation to pre‐T cells of OT as a self‐peptide of the neurohypophysial family. The selective advantage of this form of MHC‐I presentation of OT is that it would not be tightly restricted by MHC‐I alleles and would allow presentation of the OT cyclic structure. The behavior of thymic OT as a self‐antigen targeted to TEC plasma membrane was further supported by the stimulation in the production by cultured human TECs of interleukin‐6 (IL‐6) and leukemia inhibitory factor through the immunological recognition of OT by specific OT‐specific monoclonal antibodies.^42^ This study suggests that deadly self‐recognition in the thymus could lead to some cytokine release helpful for the survival and development of thymocytes that do not recognize self‐peptides.

**Figure 1:**
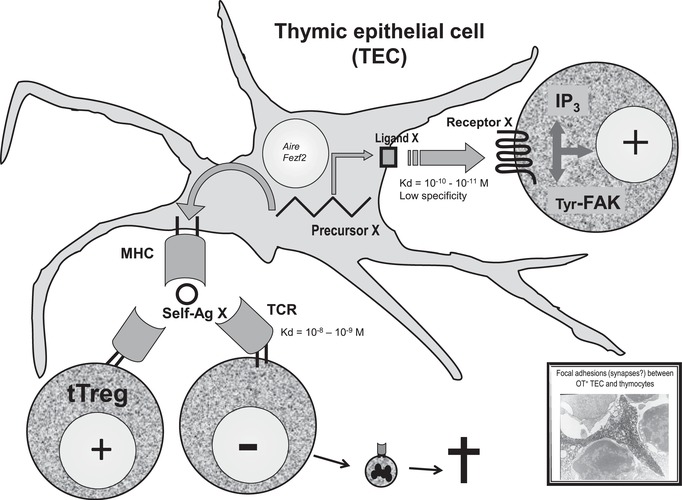
The triple role of thymic neuroendocrine self‐peptides in T cell differentiation. Following its transcription under AIRE (or FEZF2) control in TEC, a neuroendocrine precursor X is processed according to two distinct pathways. On the one hand, it is the source of a cryptocrine ligand X that is able to bind to a neuroendocrine cognate receptor expressed by T cells, to mobilize second messengers (such as IP3), and to induce intracellular events (such as phosphorylation of the focal adhesion‐related kinases p125^Fak^ and p130^Cas^ for thymic OT). This constitutes a positive accessory signal during T cell development. On the other hand, the same precursor X is also processed as the source of self‐peptide(s) X that is presented by MHC proteins of TECs or after transfer to thymic DCs. During fetal life, self‐presentation induces negative selection of T cells that are randomly bearing a TCR specific of this MHC−self‐peptide X complex. Mainly after birth, self‐presentation also promotes the generation of tT_reg_ cells specific to the same complex. The electron microscopy photograph is a generous gift from Martin Wiemann, now at the University of Duisburg‐Essen.

The hypothesis that a thymic neuropeptide actually behaves as a self‐peptide was further investigated with NT, a 13‐amino acid linear neuropeptide. Intrathymic MHC‐I presentation of NT was demonstrated according to the biochemical rules of MHC‐I presentation as established by Hans‐Georg Rammensee's laboratory in Tübingen.^43^ Interestingly, the C‐terminal sequence of NT includes tyrosine, leucine, and isoleucine residues, which can be used for anchorage to most of MHC‐I proteins.^44^ So, NT and NT‐derived C‐terminal fragments could serve as natural ligands for a majority (if not all) of MHC‐I alleles, thus also without any tight allelic restriction that is hardly conceivable for the establishment of central immune self‐tolerance to a universal peptide. This hypothesis also concords with the high degree of conservation of NT‐related C‐terminal region during evolution. All these studies allowed us to propose a theoretical model that completely transposes at the molecular level the function of the thymus in T cell development and central self‐tolerance. This was extensively discussed in Refs. 37 and 38 (Fig. 1).

In the early 2000s, Bruno Kyewski, Jens Derbinski, and Ludger Klein in Heidelberg further proposed that promiscuous gene expression of tissue‐restricted antigens (TRAs) in the thymus was a novel key to understand immune self‐tolerance and autoimmunity.^45^, ^46^ Contrary to neuroendocrine self‐peptides, TRAs do not behave as accessory signals binding to cognate receptors during T cell development. Also, while neuroendocrine self‐peptides are synthesized in all TECs, promiscuous gene expression of TRAs in the thymus is a unique property of medullary TECs (mTECs) subsets, and these genes are transiently transcribed in clusters along chromosomes.^47^ After some initial skepticism, the immunology field recognized the importance of thymus‐dependent central self‐tolerance.^37^, ^48^, ^49^ Kyewski's group also showed that thymic DCs are implicated in the presentation of TRAs synthesized in mTECs indicating a unidirectional transfer of self‐peptides inside the thymic cellular microenvironment.^50^ Patterns of promiscuous expression of insulin and casein locus‐related genes also indicated that a stochastic mechanism underlies their transcription in mTECs.^51^

The epigenetic regulation of promiscuous gene expression in the thymus is currently under intense investigation.^52^, ^53^ Most probably, these studies might explain in the future the hierarchy in the expression of neuroendocrine self‐peptides belonging to the same family, the absence of parental imprinting of *IGF2* transcription in TECs,^47^ and control of the immunological mirror of self in the thymus.

## A thymus defect as the earliest event promoting autoimmunity

**Figure 2:**
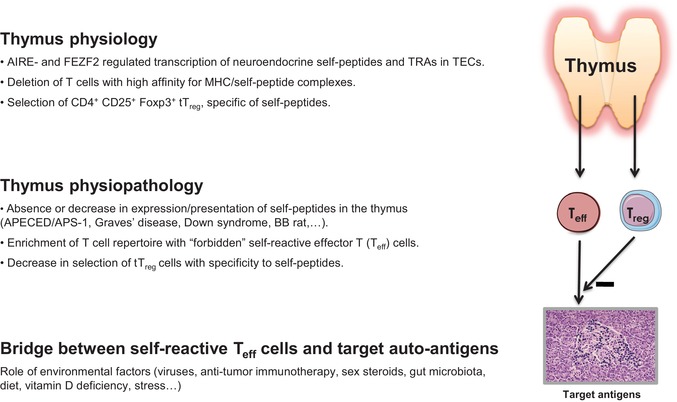
Role of the thymus in programming central self‐tolerance and in the development of autoimmunity. In normal conditions, under the control of AIRE and FEZF2, TECs express numerous genes related to neuroendocrine families or encoding TRAs. MHC presentation of these self‐peptides induces T cell differentiation, negative selection of self‐reactive T cells, and generation of tT_reg_ cells with the same specificity. In pathological conditions, the decrease in intrathymic expression and presentation of self‐peptides leads to continuous generation in the blood of self‐reactive “forbidden” T cells (T_eff_ cells), as well as to a decrease in the generation of self‐specific tT_reg_ cells. This is a condition, necessary but not sufficient, for the development of an autoimmune response against target antigens. For the clinical manifestations of an autoimmune disease, the intervention of environmental factors is also requested.

While the unique physiological function of the thymus in programming central self‐tolerance is now well established, more and more experimental observations point to a disturbance of thymus‐dependent tolerance as a key event in the early development of organ‐specific autoimmune diseases (Fig. 2). In 1973, Burnet already predicted that “forbidden” T cell clones having mutated from a state of self‐tolerance to self‐reactivity could be a major event in the physiopathology of autoimmunity.^54^ In the BioBreeding (BB) rat, an animal model of type 1 diabetes mellitus (T1D), removal of the thymus at birth prevents the appearance of autoimmune diabetes.^55^ Transplantation of the thymus from diabetes‐resistant (DR) to diabetes‐prone (DP) BB rats also inhibits this spontaneous disease.^56^ In the nonobese diabetic (NOD) mouse, another animal model of T1D, grafts of NOD thymuses to DR mouse strains induce the occurrence of autoimmune diabetes in recipients.^57^ Similarly, transplantation of embryonic NOD thymic epithelium to C57BL/6 athymic mice induces in recipients autoimmune sialitis and insulitis similar to those observed in adult female NOD.^58^ Other authors also reported defects of central tolerance and apoptosis of self‐reactive T cells in the NOD thymus.^59^, ^60^

Several groups then addressed the fundamental question whether inhibition or a significant decrease in intrathymic self‐presentation could result in a continuous release of “forbidden” self‐reactive T cells accumulating in the peripheral repertoire, as well as to a decrease in the generation of self‐reactive tT_reg_ cells. If this hypothesis was verified, it would be clear that a defect in thymic tolerogenic function might determine early the development of organ‐specific autoimmune diseases. Our laboratory investigated this question more particularly for the pathogenesis of autoimmune insulin‐dependent T1D. Concerning the members of the insulin gene family, *IGF2* is highly expressed in TECs in the cortex and medulla of the thymus from different species. *IGF1* transcripts are detected in all TECs as well as in thymic macrophages, while *INS* is transitorily transcribed in rare subsets of mTECs.^47^ This hierarchical profile implicates that IGF‐2 is highly tolerated by the T cell system since T cell tolerance is directly proportional to the intrathymic concentration of self‐peptides.^61^ Using fetal thymic organ cultures, we also observed that T cell proliferation and differentiation was severely inhibited after the interference of IGF‐mediated signaling between TECs and thymocytes, an effect that was absent after the blockade of proinsulin signaling.^62^ *Igf2* transcription is detected in the thymus of BB‐DR rats but is absent in the thymus of ±85% of BB‐DP rats, in close coincidence with the incidence of autoimmune diabetes in BB‐DP rats.^63^ These data may explain the BB‐DP lymphopenia (including a decrease in the frequency of rat T_reg_ cells), as well as defective programming of central tolerance to insulin‐secreting islet β cells.

A specific topography and hierarchy also exist in the expression of the two mouse insulin genes, *Ins1* and *Ins2*. *Ins2* transcription predominates in murine mTECs, while *Ins1* is mostly expressed in islet β cells. These contrasted profiles explain why insulitis and autoimmune diabetes are accelerated in *Ins2* ^−/−^ NOD mice,^64^ whereas these processes are significantly inhibited in *Ins1* ^−/−^ NOD mice.^65^ Levels of *Ins2* transcription in the thymus also modulate insulin‐specific T cell tolerance.^66^ Interestingly, thymic *Ins2* transcription is independent of glycemia and is markedly enhanced by an anti‐lymphotoxin‐β monoclonal antibody.^67^ However, the insulin‐specific transactivator *Mafa* also promotes thymic *Ins2* transcription and *Mafa* inactivation was shown to reduce thymic *Ins2* expression and to stimulate in parallel the generation of autoantibodies against anti‐islet β cells.^68^ *INS* transcripts are quantified at a lower level in the thymus of human fetuses with the genetic marker insulin‐dependent diabetes mellitus 2 (*IDDM2*) of susceptibility to T1D.^69^, ^70^

The identification of the autoimmune regulator gene (*AIRE*), a member of the zinc‐finger gene family, played a major role in further evidencing the central role played by a thymus dysfunction in the pathogenesis of organ‐specific autoimmunity.^71^ *AIRE* mutations determine a rare recessive congenital syndrome called autoimmune polyglandular syndrome type 1 or autoimmune polyendocrinopathy‐candidiasis‐ectodermal dystrophy syndrome.^72^ *Aire* transcription is maximal in mTECs, and *Aire^−/−^* mice develop several autoimmune processes in parallel with a marked decrease in the intrathymic expression of numerous neuroendocrine self‐peptides (including OT, INS, IGF‐2, and NPY) and many TRAs.^73^ Both *IDDM2* and *AIRE* transcription determine the level of *INS* transcription in the human thymus.^74^ The development and differentiation of murine *Aire*‐expressing mTECs is regulated by RANK signals from thymic CD4^+^CD3^−^ lymphoid tissue inducer cells.^75^ Interestingly, extrathymic *Aire*‐expressing cells were recently identified as distinct bone marrow−derived tolerogenic cells that could anergize in secondary lymphoid organs effector self‐reactive CD4^+^ T cells having escaped thymic negative selection.^76^, ^77^

More recently, it has been shown that the gene *Fezf2* encoding fasciculation and elongation protein zeta family zinc finger‐2 protein also controls intrathymic transcription of self‐peptides, the majority of which are not regulated by *Aire*. *Fezf2* disruption is also associated with autoimmune processes in the periphery.^78^ Noteworthy, *Fezf2* is also a transcription factor implicated in some developmental processes of the central nervous system (CNS).

With regard to the most frequent autoimmune endocrine disease, all major thyroid‐specific autoantigens, such as thyroperoxydase, thyroglobulin, and thyrotropin receptor (TSHR), are also transcribed in human TECs in normal conditions.^79^, ^80^, ^81^ It was further shown that homozygotes for an SNP allele predisposing to Graves’ disease have significantly lower intrathymic *TSHR* transcripts than carriers of the protective allele.^82^

A defect in α‐myosin expression in TECs exerts a major role in the physiopathology of autoimmune myocarditis,^83^ and a defect in Aire‐mediated central tolerance to myelin protein zero promotes the appearance of an autoimmune T_H_1 effector response toward peripheral nerves.^84^ There is also large supportive evidence that thymic tolerance plays an essential role in CNS autoimmune diseases.^85^

## Coevolution of immune and neuroendocrine systems

**Figure 3:**
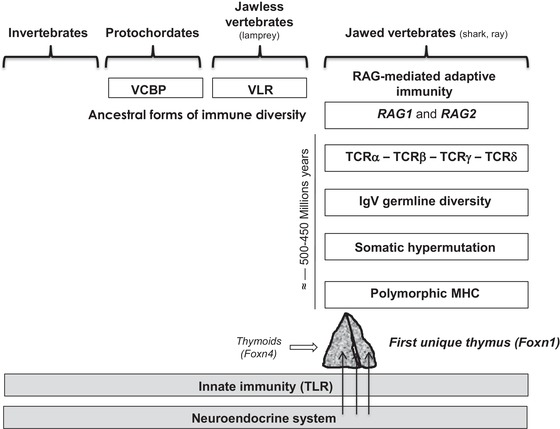
Integrated evolution of the immune and neuroendocrine systems. Neuroendocrine precursors did not evolve extensively except by gene duplication and differential RNA splicing. Throughout evolution, the neuroendocrine and innate immune system have evolved in parallel, and still coexist in all living species without any aggression of the innate immune system toward neuroendocrine glands. A high risk of inherent autoimmunity toward neuroendocrine tissues resulted from the appearance of recombination‐activating genes *RAG1* and *RAG2*, and RAG‐dependent adaptive immunity in jawed cartilaginous fishes some 450 million years ago. Preceded by ancestor paralog *Foxn4*‐expressing thymoids in gill baskets of lamprey larvae, the first unique thymus (with *Foxn1*‐expressing TECs) also emerged in jawed vertebrates. The intrathymic presentation of dominant neuroendocrine self‐peptides (arrows) may be viewed *a posteriori* as a very efficient and economical way to instruct the adaptive T cell system in recognizing and tolerizing neuroendocrine families already during thymus‐dependent T cell differentiation in fetal life. VCBP, variable region‐containing chitin‐binding protein; VLR, variable lymphocyte receptor.

The innate immune system evolves in parallel with the neuroendocrine system in all living species without any sign of autotoxicity/autoimmunity (Fig. 3). Toll‐like receptors, which are major mediators of innate immunity, do not react against normal or undamaged self. Primitive forms of immune diversity are present in agnathans, mediated by diverse variable lymphocyte receptors, with 4−12 leucine‐rich repeat modules that were most probably assembled by some gene conversion process.^86^ About 450–500 million years ago, the emergence of transposon‐like recombination–activating genes *Rag1* and *Rag2* in jawed fishes was responsible for the appearance of a novel and highly complex system of immune defenses, the adaptive immunity. The subsequent development of the combinatorial immune system has been sometimes regarded as the immunological “Big Bang.” Because of its high risk of autotoxicity inherent to its diversity, the emergence of adaptive immunity exerted an evolutionary pressure so strong that, according to Ehrlich's prediction of *horror autotoxicus*, novel structures and mechanisms appeared with the specific role of orchestrating immune self‐tolerance. The first unique thymus also appeared in sharks and rays but it was preceded by thymoid lymphoepithelial structures located in the gill baskets of lamprey larvae.^87^ These structures express the gene encoding forkhead box N4 (*Foxn4*), the paralog of *Foxn1*, which is responsible for the differentiation of the thymic epithelium in most vertebrates. Thus, *Foxn4*/*Foxn1* determined the emergence of the thymic epithelium, which is an absolute requirement for T cell differentiation and programming of central immune self‐tolerance.^88^

The hierarchy observed in the organization of the thymic repertoire of neuroendocrine self‐peptides has also evolutive implications. Neuroendocrine functions having been installed before adaptive immunity, they had to be protected against autoimmunity. OT is implicated at different steps of the reproductive process and is therefore fundamental for the preservation of animal and human species. Because of its predominance in the thymus, OT is much more tolerated than VP, its neurohypophysial homolog, which mainly regulates water homeostasis and vascular pressure. This lower immunological tolerance of VP may explain why rare cases of autoimmune central diabetes insipidus have been repeatedly observed.^89^

In the insulin family, insulin is the primary T1D autoantigen and no autoimmunity has been reported against IGF‐2, which is fundamental for fetal growth and development. Nevertheless, because of their homology, thymic neuroendocrine self‐peptides program immune cross‐tolerance to their whole family, and tolerance of insulin is indeed decreased in *Igf2^−/−^* mice.^90^

## Conceptual translation: from immunogenic to tolerogenic vaccines

Until now, the development of anti‐infective vaccines was essentially based on immunogenic and memory properties of the adaptive immune system. Future vaccines against autoimmune diseases could be developed on the basis of the initial property of self‐tolerance programmed for this system and recent knowledge about the potent tolerogenic properties of the thymus, which have not been exploited until now. As reported above, although *Ins2* is expressed at very low levels in rare mTEC subsets, proinsulin per se does not exert any tolerogenic properties that could be used to reprogram immunological tolerance toward islet β cells. With the exception of only two studies (that were not confirmed until now),^91^, ^92^ all the clinical trials based on insulin failed to protect the residual β cell mass from the diabetogenic autoimmune response. At the opposite, the potent immunogenic properties of insulin were repeatedly evidenced,^93^, ^94^ and insulin immunogenicity could actually be linked to the very low level of *INS* transcription in mTEC subsets. The risk of hypersensitivity or anaphylaxis following administration of an autoantigen was also reported.^95^

**Figure 4:**
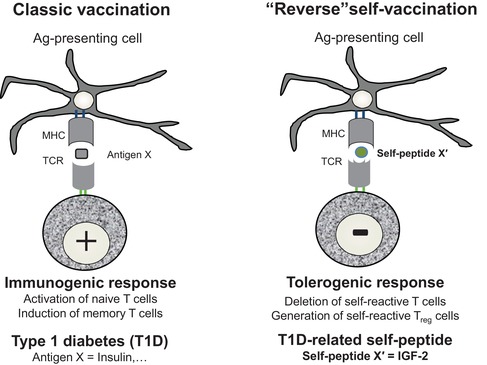
Classical vaccination and the concept of “reverse” self‐vaccination. Classical vaccination essentially relies on an immunogenic response (naive T cell activation and induction of memory immune cells) elicited by administration of antigen(s) representative of pathogens. T1D pathogenesis also relies on an immunogenic response targeting several T1D antigens, such as insulin as the primary T1D autoantigen (X). The novel type of “reverse” self‐vaccination proposes to use thymus self‐peptides for promoting a tolerogenic response (deletion of self‐reactive T cells and generation of self‐reactive T_reg_ cells). For T1D prevention and cure, the corresponding thymus self‐peptide is IGF‐2 (X′). Noteworthy, insulin is actually an “altered self” peptide of IGF‐2.^101^^−^ 103

IGF‐2 could provide a more efficient basis than insulin for developing a specific “reverse/tolerogenic self‐vaccination”^96^ (Fig. 4) based on the following data:

- *Igf2* is a dominant member of the insulin family expressed in the thymus.^35^
- *Igf2* transcription is defective in the thymus of BB‐DP rats.^63^
- IGF‐2 B_11−25_ and insulin B9−23 (InsB_9−23_) compete for binding to DQ2 and DQ8 (collaboration with K. Wücherpfennig, unpublished data), the MHC‐II alleles conferring the highest genetic susceptibility to T1D.
- Contrary to InsB_9−23_, IGF‐2 B_11−25_ presentation by peripheral blood mononuclear cells isolated from DQ8^+^ diabetic patients induces a tolerogenic cytokine profile with marked IL‐10 induction.^97^
- IGF‐2 mediates significant cross‐tolerance to insulin.^90^
- Infection of a murine TEC line with the diabetogenic coxsackievirus B4‐E2 inhibits *Igf2* transcription and IGF‐2 production.^98^
- IGF‐2 induces the activation of T_reg_ and B_reg_ cells.^99^, ^100^

This promising concept of reverse tolerogenic self‐vaccination is under current development with the active support of Wallonia DGO6 Win2Wal THYDIA Project.

## Conclusion

Far from being a useless “vestigial” organ, numerous studies performed worldwide have definitively demonstrated that the thymus is crucial for ensuring the global homeostasis of the adaptive immune system and is unique in programming central immune self‐tolerance. Presentation of self‐peptides in the thymus is the basic mechanism that underlies T cell differentiation, negative selection of self‐reactive T cell clones, and generation of tT_reg_ cells. There is no doubt that thymus‐based novel strategies could alleviate in the future the weight of so many known and still unknown autoimmune diseases that remain the heavy tribute, mainly paid by mankind, for the performance and extreme diversity of its highly complex adaptive immunity.

## Competing interests

The authors declare no competing interests.
